# From Cost to Value: A Comparative Analysis of Medical and Surgical Management of Inflammatory Bowel Disease in a Public Referral Hospital in Mexico

**DOI:** 10.7759/cureus.106153

**Published:** 2026-03-30

**Authors:** Angie Eleoneai Vargas Rodríguez, Samanta Kin Dosal Limón, Raquel Yazmin López Pérez, Paola Rosales Téllez, Billy Jiménez Bobadilla, Alma Rosa Sánchez Conejo, Bertha Alicia Mancilla Salcedo, Alfonso David Aparicio Bolaños, Jorge Luis De León Rendón

**Affiliations:** 1 Surgery, Hospital General de México Dr. Eduardo Liceaga, Mexico City, MEX; 2 Medicine, Universidad Nacional Autónoma de México, Mexico City, MEX; 3 Gastroenterology, Hospital General de México Dr. Eduardo Liceaga, Mexico City, MEX; 4 Cardiology, Hospital General de México Dr. Eduardo Liceaga, Mexico City, MEX; 5 Health Policy, Hospital General de México Dr. Eduardo Liceaga, Mexico City, MEX; 6 Health Policy, KPMG AG Wirtschaftsprüfungsgesellschaft, Stuttgart, DEU; 7 Colorectal Surgery, Hospital General de México Dr. Eduardo Liceaga, Mexico City, MEX; 8 Medicine, School of Medicine, Faculty of Health Sciences, , Universidad Anáhuac México, Mexico City, MEX

**Keywords:** colorectal surgery, crohn disease, healthcare costs, inflammatory bowel disease, medical management, public hospital, real-world data, surgical management, ulcerative colitis

## Abstract

Background and objective: Inflammatory bowel disease (IBD) represents a growing clinical and economic challenge for healthcare systems, particularly in settings that provide free access to specialised care. Advances in medical therapy have modified disease outcomes but have also reshaped healthcare expenditure, while surgical management continues to represent high-impact cost events. Integrated analyses comparing medical and surgical costs from the perspective of public healthcare systems remain scarce in Latin America. This study aims to compare the economic burden of medical versus surgical-hospital management in patients with IBD by quantifying cumulative direct costs over 12 months from an institutional perspective using real-world data.

Patients and methods: A retrospective observational study using real-world data was conducted at the Inflammatory Bowel Disease Clinic of the Coloproctology Service, Hospital General de México “Dr. Eduardo Liceaga," a tertiary-level public referral hospital in Mexico City. A total of 118 patients (100%) with confirmed IBD managed over 12 months were included. Patients were categorized according to medical or surgical-hospital management. Direct costs related to pharmacological treatment, hospitalization, and surgical care were quantified from an institutional perspective and analyzed descriptively.

Results: Among the 118 patients included (100%), 95 (80.5%) had ulcerative colitis and 23 (19.5%) had Crohn’s disease. Medical management was required in 91 patients (77.1%), including advanced therapies in 27 (22.9%). Surgical-hospital management was necessary in 24 patients (20.3%). The cumulative annual cost of medical management for the cohort was Mexican peso (MXN) 16.4 million. In contrast, surgical-hospital management generated a total accumulated cost of MXN 15.0 million, despite involving a substantially smaller proportion of patients. Overall, approximately 95% of direct healthcare costs were absorbed by the public institution within a free-access care model.

Conclusions: In a public referral hospital, the economic burden of IBD reflects both sustained pharmacological investment and high-impact surgical events. The comparable magnitude of cumulative medical and surgical expenditures underscores the need to interpret healthcare costs in relation to clinical trajectories rather than patient volume alone, supporting value-oriented strategies to optimize resource allocation in publicly funded healthcare systems.

## Introduction

Inflammatory bowel disease (IBD), encompassing ulcerative colitis (UC) and Crohn’s disease (CD), has emerged as a growing global public health challenge. Once considered a disease of industrialized nations, IBD incidence and prevalence have increased steadily in middle- and low-income regions, including Latin America, placing increasing pressure on healthcare systems operating under universal coverage and free-access models [1,2]. As a chronic, relapsing condition affecting predominantly individuals of working age, IBD generates substantial direct medical costs, including hospitalizations, advanced pharmacological therapies, surgery, and diagnostic procedures, as well as indirect costs related to productivity loss and impaired quality of life [3,4].

In high-income countries, the economic burden of IBD has been extensively characterized, with hospital-based care, surgical interventions, and advanced therapies representing major drivers of healthcare expenditure. Although biologic agents and small molecules have transformed disease outcomes, these therapeutic advances have also led to escalating costs, raising concerns regarding long-term system sustainability [5,6]. Surgical management remains essential in selected clinical scenarios, such as refractory disease or structural complications, and is typically associated with high short-term resource utilization [7,8]. Within this context, evaluating not only costs but also the value generated by different therapeutic strategies becomes increasingly relevant.

In Latin America, economic evaluations of IBD remain limited and heterogeneous. Most available studies have focused on epidemiological or clinical characteristics, while systematic analyses of healthcare costs and resource utilization are scarce [9,10]. Emerging data from countries such as Colombia and Brazil suggest that costs increase with disease progression and therapeutic escalation, although extrapolation is limited by structural differences across healthcare systems [11,12]. In Mexico, evidence regarding institutional costs remains incipient despite documented impacts on quality of life and healthcare utilization [13].

In publicly funded healthcare systems providing free medical care, understanding the relationship between resource consumption and clinical management strategies is critical to informing efficient allocation and long-term sustainability [6,14]. Therefore, the present study aimed to evaluate the direct medical and surgical costs associated with IBD management in a public referral hospital in Mexico and to compare the relative economic implications of medical versus surgical treatment approaches within a free-care model.

## Materials and methods

Study design

A retrospective observational study with an economic analysis approach was conducted using real-world data at the Inflammatory Bowel Disease Clinic of the Coloproctology Service, Hospital General de México Dr. Eduardo Liceaga, a tertiary-level public referral hospital located in Mexico City, Mexico, operating under a publicly funded healthcare system that provides specialized care to patients with IBD within a predominantly free-access public healthcare model. A total of 118 consecutive patients with a confirmed diagnosis of IBD were included. All patients were managed at the Inflammatory Bowel Disease Clinic during a 12-month period (May 2023 to May 2024). Medical or hospital-based and surgical management was provided according to clinical indications established by the treating multidisciplinary team, without any intervention or modification to routine clinical practice.

Patients were excluded from the analysis if they had incomplete clinical or economic information in the institutional medical records, insufficient follow-up to allow adequate cost evaluation, or if their primary management had been conducted at another institution with incomplete documentation of medical or surgical interventions performed at our center. In addition, cases with an uncertain diagnosis or insufficient clinical, endoscopic, histologic, or radiologic information to confirm IBD were excluded, as were records lacking sufficient detail to accurately estimate healthcare resource utilization and associated costs.

Disease classification and activity assessment

Disease phenotype and extent were classified according to the Montreal classification system. Ulcerative colitis was categorized according to disease extent as E1 (ulcerative proctitis), E2 (left-sided colitis), and E3 (extensive colitis). Crohn’s disease was classified according to age at diagnosis as A2 (17-40 years) and A3 (>40 years), disease location as L2 (colonic disease), L3 (ileocolonic disease), and L4 (upper gastrointestinal involvement), and disease behavior as B1 (non-stricturing, non-penetrating), B2 (stricturing), and B3 (penetrating) [15].

In patients with ulcerative colitis, clinical disease activity was assessed using the Truelove and Witts criteria as part of routine clinical evaluation [16]. Endoscopic activity was evaluated using the Mayo endoscopic subscore [17]. Histological inflammatory activity was assessed using the Riley index [18].

Systematic assessment of disease activity using standardized clinical or endoscopic indices was not performed in patients with Crohn’s disease during the study period. The present analysis was primarily designed to characterize management patterns and direct medical costs rather than perform a comparative evaluation of disease activity between phenotypes.

Medical management of IBD

Medical management followed a treat-to-target strategy in accordance with the Selecting Therapeutic Targets in Inflammatory Bowel Disease (STRIDE) II consensus recommendations, prioritizing clinical and endoscopic remission and therapeutic optimization based on disease activity and treatment response [19]. Therapeutic decisions were aligned with current international clinical practice guidelines issued by the European Crohn’s and Colitis Organization (ECCO) [20,21].

Hospital management, complications, and surgical indication

Indications for hospitalization, management of complications, and surgical treatment were established according to ECCO guidelines for medical-surgical management of IBD [20-23]. Surgical intervention was indicated in cases of refractory or steroid-dependent disease, failure of optimized medical therapy, structural complications, acute severe disease, or IBD-associated dysplasia or colorectal cancer [20-23].

Economic analysis perspective

The economic analysis was conducted from the institutional perspective and considered exclusively direct medical costs. Indirect, non-medical, and intangible costs were not included. Direct medical costs related to hospital-based and surgical management included surgery, medical supplies, hospitalization, and perioperative care, estimated using the institutional cost-recovery fee schedule. Costs related to medical management included conventional and advanced pharmacological therapies. Medication costs were estimated using maximum retail prices to standardize cost comparisons. The time horizon was defined as short-term (one month) and long-term (twelve months) following initiation of medical or surgical hospital management.

Data collection and analysis

All clinical and economic variables were extracted from institutional electronic medical records and administrative cost databases and systematically recorded in a structured database designed for the study. The data collection and cost estimation processes followed standardized institutional procedures to ensure consistency and reproducibility. Statistical analyses were performed using SPSS Statistics version 27.0 (IBM Corp., Armonk, NY, USA). Continuous variables were summarized as mean ± standard deviation (SD), and categorical variables as frequencies and percentages. No inferential statistical comparisons were performed, as the study was designed as a descriptive economic evaluation.

## Results

A total of 118 patients with a confirmed diagnosis of IBD were included in the analysis. Of these, 95 patients (80.5%) had UC and 23 patients (19.5%) had CD. The baseline demographic and clinical characteristics of the cohort are summarized in Table 1. The cohort showed a predominance of female patients (70/118; 59.3%), with a mean age of 41 years (range: 18 to 75 years). Most patients (80/118; 67.8%) were diagnosed before the age of 40 years. Disease duration ranged from zero to 43 years. Among patients with UC (n=95), disease extent according to the Montreal classification demonstrated pancolitis in 55/95 patients (57.9%), left-sided colitis in 31/95 (32.6%), and proctitis in 9/95 (9.5%).

**Table 1:** Demographic and clinical characteristics of patients with IBD The Montreal classification categorizes UC according to disease extent (E1–E3) and CD according to age at diagnosis (A), disease location (L), and disease behavior (B); L1 indicates ileal disease, L2 colonic disease, L3 ileocolonic disease, and L4 upper gastrointestinal involvement; B1 indicates non-stricturing non-penetrating disease, B2 stricturing disease, and B3 penetrating disease. The letter P denotes the presence of perianal disease. Clinical activity in UC was assessed using the Truelove and Witts criteria, endoscopic activity using the Mayo endoscopic subscore, and histological inflammatory activity using the Riley index. SD: Standard deviation; IBD: Inflammatory bowel disease; UC: Ulcerative colitis; CD: Crohn’s disease

Variable	Total (n = 118)	UC (n = 95)	CD (n = 23)
Sex, n (%)			
Male	48 (40.7)	37 (38.9)	10 (43.5)
Female	70 (59.3)	58 (61.1)	13 (56.5)
Age, mean ± SD (range)	41 ± 14 (18–75)	41 ± 13 (18–75)	42 ± 15 (19–74)
Age at diagnosis <40 years, n (%)	80 (67.8)	65 (68.4)	15 (65.2)
Disease duration, years (range)	0-43	1-3	1-21
Positive smoking history, n (%)	24 (20.3)	14 (14.7)	10 (43.5)
Montreal classification – UC, n (%)			
Proctitis (E1)	—	9 (9.5)	—
Left-sided colitis (E2)	—	31 (32.6)	—
Pancolitis (E3)	—	55 (57.9)	—
Montreal classification – CD, n (%)			
Age at diagnosis (A)			
A1 (≤16 years)	—	—	0 (0)
A2 (17–40 years)	—	—	14 (60.9)
A3 (>40 years)	—	—	9 (39.1)
Location (L)			
L1 (Ileal)	—	—	3 (13.0)
L2 (Colonic)	—	—	9 (39.1)
L3 (Ileocolonic)	—	—	10 (43.5)
L4 (Upper gastrointestinal tract)	—	—	1 (4.3)
Disease behavior (B)			
B1 (Non-stricturing, non-penetrating)	—	—	6 (26.0)
B2 (Stricturing)	—	—	15 (65.2)
B3 (Penetrating)	—	—	2 (8.6)
Perianal disease (P)	—	—	6 (26.1)
Clinical activity in UC, n (%)			
Inactive	—	74 (77.9)	—
Mild	—	19 (20.0)	—
Moderate	—	2 (2.1)	—
Severe	—	0 (0)	—
Endoscopic activity, n (%)			
Inactive	—	7 (7.4)	—
Mild	—	32 (33.7)	—
Moderate	—	36 (37.9)	—
Severe	—	20 (21.1)	—
Histological activity, n (%)			
Inactive	—	10 (10.5)	—
Mild	—	43 (45.3)	—
Moderate	—	28 (29.5)	—
Severe	—	14 (14.7)	—
Extraintestinal manifestations, n (%)			
Arthralgia/arthritis	40 (33.9)	28 (29.7)	12 (52.0)
Primary sclerosing cholangitis	30 (25.4)	23 (24.2)	7 (30.4)
Spondylitis	1 (0.85)	1 (1.1)	0 (0)
Pyoderma gangrenosum	1 (0.85)	1 (1.1)	0 (0)
Uveitis	1 (0.85)	1 (1.1)	0 (0)
Medical treatment, n (%)			
Conventional therapy	91 (77.1)	80 (84.2)	11 (47.8)
Advanced therapy	27 (22.9)	15 (15.8)	12 (52.2)
Surgical treatment, n (%)			
Yes	24 (20.3)	15 (15.8)	9 (39.1)
No	94 (79.7)	80 (84.2)	14 (60.9)

In the CD subgroup (n=23), most patients were classified within age category A2 (14/23; 60.9%), followed by A3 (9/23; 39.1%). Regarding disease location, ileocolonic involvement (L3) was identified in 10/23 patients (43.5%), colonic disease (L2) in 9/23 (39.1%), ileal disease (L1) in 3/23 (13.0%), and upper gastrointestinal involvement (L4) in 1/23 (4.3%). In terms of disease behavior, stricturing disease (B2) was observed in 15/23 patients (65.2%), non-stricturing non-penetrating disease (B1) in 6/23 (26.0%), and penetrating disease (B3) in 2/23 (8.6%). Perianal disease was documented in 6/23 patients (26.1%).

Clinical disease activity among patients with UC, assessed using the Truelove and Witts criteria [16], showed inactive disease in 74/95 patients (77.9%), mild activity in 19/95 (20.0%), and moderate activity in 2/95 (2.1%). No patients met the criteria for severe clinical activity. Endoscopic activity evaluated using the Mayo endoscopic subscore showed inactive disease in 7/95 patients (7.4%), mild activity in 32/95 (33.7%), moderate activity in 36/95 (37.9%), and severe activity in 20/95 (21.1%). Histological activity assessed using the Riley index demonstrated inactive disease in 10/95 patients (10.5%), mild activity in 43/95 (45.3%), moderate activity in 28/95 (29.5%), and severe activity in 14/95 (14.7%).

**Figure 1:**
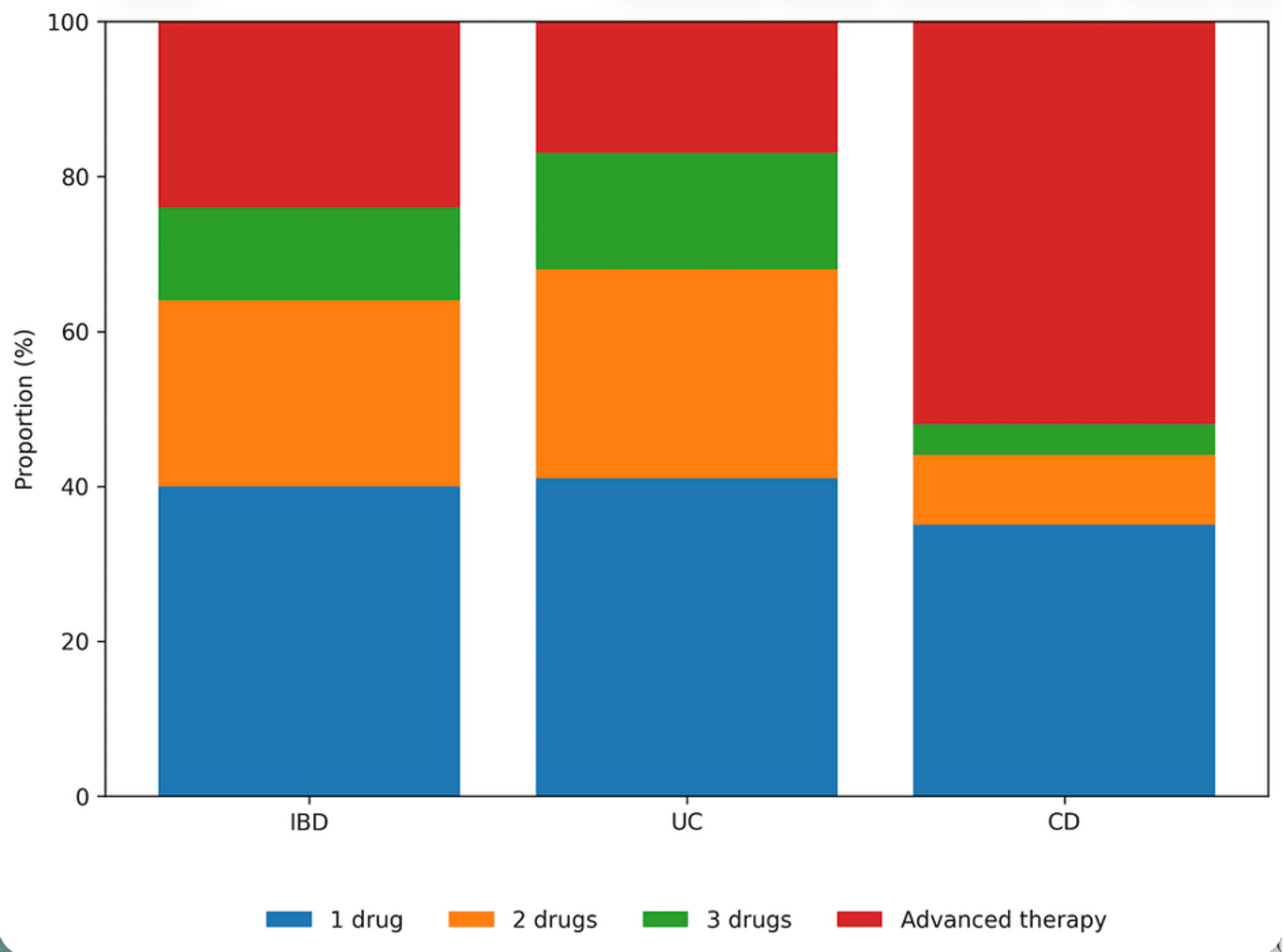
Percentage distribution of medical treatment regimens in patients with IBD, UC, and CD This graph shows the proportional distribution of therapeutic regimens used in the overall IBD cohort and stratified by UC and CD. Four categories are included: treatment with one conventional drug, combination therapy with two conventional drugs, combination therapy with three conventional drugs, and advanced therapy. IBD: Inflammatory bowel disease, UC: Ulcerative colitis, CD: Crohn's disease

Extraintestinal manifestations were identified in 40/118 patients (33.9%). The most frequently documented manifestations included arthralgia or peripheral arthritis and primary sclerosing cholangitis. The detailed distribution of extraintestinal manifestations is presented in Table 1. Regarding therapeutic management, 91/118 patients (77.1%) received conventional medical therapy, whereas 27/118 patients (22.9%) were treated with advanced therapies during the study period. The distribution of treatment regimens is illustrated in Figure 1.

Surgical or hospital-based management was required in 24/118 patients (20.3%). Surgical interventions were performed in 9/23 patients with CD (39.1%) and in 15/95 patients with UC (15.8%). In patients with UC, the most common indication for surgery was lack of response to medical therapy. In CD, surgical indications included structural complications such as intestinal strictures and fistulizing disease. Surgical procedures included total or subtotal colectomy in UC and intestinal resections or perianal procedures in CD, as summarized in Table 1.

The economic analysis of medical management is summarized in Table 2. The mean monthly cost per patient receiving medical treatment was MXN 11,612.45, corresponding to an estimated annual cost of MXN 139,349.40 per patient. For the entire cohort of 118 patients, the total cost of medical management during the 12-month study period was MXN 16,443,229.20. Of this amount, MXN 15,621,067.74 (approximately 95%) corresponded to costs absorbed by the institution, while the remaining 5% corresponded to costs not absorbed by the institution.

**Table 2:** Direct healthcare costs of medical and surgical management in patients with IBD All costs are expressed in MXN. Medical management costs include both conventional pharmacological therapy and advanced therapies. Total costs correspond to cumulative healthcare expenditure during the study period. 'Cost absorbed by the institution' represents the proportion of the publicly funded healthcare expenditure directly covered by our institution (95%). IBD: Inflammatory bowel disease, MXN: Mexican pesos

Variable		Cost (MXN)
Medical management	Average monthly cost per patient	11,612.45
	Average annual cost per patient	1,39,349.40
	Total cost of medical management	1,64,43,229.20
	Cost absorbed by the institution (95%)	1,56,21,067.74
Surgical-hospital management	Mean cost per surgical event	6,26,847.45
	Total cost of surgical management	1,50,44,339.00
	Cost absorbed by the institution (95%)	1,42,92,121.86
Total healthcare cost	Total cost (medical + surgical)	3,14,87,568.00
	Total cost absorbed by the institution	2,99,13,189.60

Among the cohort, 91 patients (77.1%) received conventional therapy and 27 patients (22.9%) received advanced therapies. Costs associated with surgical-hospital management are also presented in Table 2. The mean cost per surgical-hospital event was MXN 626,847.45. During the study period, 24/118 patients (20.3%) required surgical intervention, resulting in a total accumulated cost of MXN 15,044,339.00. Of this amount, MXN 14,292,121.86 (approximately 95%) corresponded to institutional cost absorption, while the remaining 5% corresponded to costs not absorbed by the institution.

At the time of data collection, 112/118 patients (95%) treated at the IBD Clinic received free medical care under the institutional care model. The remaining 6/118 patients (5%) corresponded to individuals insured by other public healthcare systems, including the Mexican Institute of Social Security (IMSS) or the Institute for Social Security and Services for State Workers (ISSSTE), who were treated at the institution through interinstitutional referral arrangements. Because these patients did not receive full institutional therapeutic coverage, particularly regarding medication supply, the costs associated with their pharmacological treatment were not included in the economic analysis.

Overall, the analysis describes the clinical profile, management patterns, and direct medical costs associated with the care of patients with IBD treated at a tertiary public referral center in Mexico. Medical management represented the predominant therapeutic strategy, while surgical-hospital interventions were required in a smaller proportion of patients. The economic evaluation provides a detailed description of direct medical costs associated with both treatment approaches within the institutional care model.

## Discussion

Inflammatory bowel disease represents an increasing challenge for healthcare systems due to its chronic course and the progressive economic burden associated with long-term management [1,2]. Over the past decade, the cost structure of IBD has shifted, with an increasing proportion of healthcare expenditure attributable to pharmacological therapy, particularly advanced treatments, rather than hospitalization or surgery alone [3-7,12,14]. In this context, the present study provides real-world evidence from a public referral hospital in Mexico where most patients receive care with full institutional coverage, allowing estimation of the economic burden directly absorbed by the healthcare system.

A key finding of this analysis is that the cumulative cost of medical management in the overall cohort (n = 118, 100%) (≈ MXN 16.4 million) was comparable to the total cost generated by surgical-hospital care in a much smaller subgroup of patients who required surgery (n = 24, 20.3%) (≈ MXN 15 million). Despite the lower number of surgical cases, the high cost per surgical event resulted in a substantial concentration of healthcare expenditure. When considering only costs absorbed by the institution, the overall pattern remained consistent.

These findings illustrate two complementary economic dynamics in IBD care. Pharmacological therapy represents a sustained and cumulative expenditure associated with long-term disease control, whereas surgery corresponds to episodic events with high immediate financial impact. From a health system perspective, this pattern highlights the potential value of strategies aimed at early inflammatory control and prevention of complications that may lead to high-cost surgical interventions [6,7,14].

The increasing use of advanced therapies has substantially modified the economic profile of IBD management worldwide [5,6,12]. However, this phenomenon should be interpreted within the context of disease biology. Persistent inflammation may lead to cumulative intestinal damage and progression toward stricturing or penetrating phenotypes, increasing the risk of hospitalization or surgery [2,18,20,21]. Economic models suggest that early inflammatory control may reduce high-cost events over time [12,14,24,25], although the present analysis was descriptive and did not incorporate formal cost-effectiveness modeling.

Importantly, surgery in IBD should not be interpreted as therapeutic failure. In several clinical scenarios, including structural complications, refractory disease, dysplasia or neoplasia, and complex perianal disease, it constitutes the standard of care according to ECCO guidelines and ECCO-European Society of Colo-Proctology (ESCP) consensus statements [20-23]. Although surgical interventions involve high initial expenditure, they may provide sustained clinical benefit in selected patients [8,25,26]. Furthermore, surgical management may be associated with postoperative complications, disease recurrence, and impacts on quality of life and productivity, factors that contribute to the overall societal cost of the disease [27,28].

The pattern observed in this study also reflects the context of therapeutic escalation during a period of institutional transition. The analyzed period corresponds to the first year in which advanced therapies became formally available within our institutional public healthcare system. Consequently, the relatively limited proportion of patients receiving these therapies likely reflects early implementation and strict selection criteria. Strategies such as appropriate patient selection, early assessment of treatment response, and therapeutic drug monitoring may help optimize treatment efficiency and reduce unnecessary therapeutic escalation [24,29].

Although treat-to-target strategies have demonstrated improved clinical outcomes [19], the present study was not designed to evaluate their economic impact. Additionally, costs associated with laboratory monitoring, biomarkers, imaging studies, and outpatient care were not included, which may lead to an underestimation of the total economic burden. Nevertheless, available evidence suggests that early initiation of effective therapy within a potential 'window of opportunity' may modify disease trajectory and reduce cumulative intestinal damage [21,30].

A distinctive feature of this study is the publicly funded context of care delivery. Approximately 95% of healthcare expenditures were absorbed by our institution, highlighting the magnitude of the institutional financial commitment and the importance of value-oriented strategies in healthcare systems providing universal or near-universal access to care.

This study has several methodological strengths. It provides real-world evidence from a tertiary-level public referral center, reflecting clinical practice within a predominantly free-access healthcare system. The use of an institutional perspective allows direct estimation of healthcare costs absorbed by the public system. Additionally, the comparative evaluation of medical and surgical-hospital management provides a comprehensive view of cost distribution across distinct clinical pathways.

This study has several important limitations that should be acknowledged. These include its retrospective design, the one-year time horizon, and the absence of formal cost-effectiveness modeling. Indirect costs such as productivity loss were not included, which may underestimate the overall economic impact. Furthermore, systematic assessment of disease activity in patients with Crohn’s disease was not available for all individuals during the study period.

The economic burden of IBD in a public referral hospital reflects the coexistence of sustained pharmacological expenditure and high-impact surgical events. These findings highlight the importance of integrated clinical and economic strategies aimed at early disease control, prevention of complications, and optimization of healthcare resource utilization in publicly funded healthcare systems.

## Conclusions

The analysis conducted in a public referral hospital demonstrates that the economic burden of IBD is distributed between sustained pharmacological investment and high-impact surgical-hospital events within a healthcare system where care is predominantly free, and costs are absorbed by the public sector. The comparable magnitude between the annual accumulated expenditure of medical management (MXN 16.4 million) and the concentrated cost of the surgical subgroup (MXN 15.0 million) highlights that cost should not be interpreted in isolation but rather as the financial expression of distinct clinical trajectories.

Moving from cost to value requires recognizing that sustainability does not rely solely on reducing expenditure but on optimizing resource allocation through strategies focused on patient stratification, timely inflammatory control, and complication prevention. In public health systems with universal or near-universal coverage, this approach is essential to balance therapeutic investment, institutional efficiency, and quality of care. Prospective studies incorporating formal economic evaluations will be necessary to more precisely define the impact of these strategies across longer time horizons.
